# Have the Chinese Older Adults Received Adequate Healthcare Services since the 2009 Health Reform? Examining the Socioeconomic Disparity in Receiving Adequate Healthcare

**DOI:** 10.3390/healthcare8030208

**Published:** 2020-07-10

**Authors:** Yen-Han Lee, Yen-Chang Chang, Yun-Ting Wang, Mack Shelley

**Affiliations:** 1 Department of Applied Health Sciences, School of Public Health, Indiana University, Bloomington, IN 47405, USA; 2 Center for General Education, National Tsing Hua University, Hsinchu City 300, Taiwan; yenchang@mx.nthu.edu.tw; 3 Department of Nursing, Taipei Veterans General Hospital, Taipei 112, Taiwan; kitty850916@gmail.com; 4 Department of Political Science, Department of Statistics, Iowa State University, Ames, IA 50011, USA; mshelley@iastate.edu

**Keywords:** China, adequate healthcare services, older adults, socioeconomic status, social disparity

## Abstract

China launched a comprehensive health reform in 2009, as part of the central government’s plan to improve its healthcare system. This study investigates the associations of socioeconomic status with receiving adequate healthcare services among Chinese older adults following the 2009 health reform. Using the 6th and the 7th waves of the Chinese Longitudinal Healthy Longevity Survey (CLHLS), a repeated cross-sectional study design was adopted (*n* = 9305). Firth’s logistic regression models were used for statistical analysis. In the fully adjusted model, being non-married was negatively associated with adequate healthcare services (adjusted odds ratio (AOR) = 0.68, 95% confidence interval (CI): 0.54, 0.86). Higher levels of income were positively associated with adequate healthcare services (all *p*s < 0.05). Participants who relied on non-urban social insurance plans all had lower odds of receiving adequate healthcare services (all *p*s < 0.01), compared with older adults who used the urban employment basic medical insurance (UEBMI). However, disparities regarding education and urban-rural differences were not observed in the full model. As China is pushing for further reforms, vulnerable population groups, such as non-married or more impoverished older adults, should be assisted in receiving adequate healthcare services.

## 1. Introduction

Receiving adequate yet necessary healthcare services is a right for each individual. As China is pushing to achieve a comprehensive universal healthcare system [1], all citizens need to have adequate healthcare services when they need them. Between the 1960s and 1970s, the Chinese central government promoted both “barefoot doctors” and the rural cooperative medical scheme (CMS) for providing basic healthcare services to Chinese residents [1]. However, with the collapse of CMS and the diminishing role of the “barefoot doctors” in the 1980s, the Chinese residents suddenly lost their convenient yet affordable basic healthcare services. Without these services, Chinese residents suffered from financial hurdles to receive healthcare; thus, many sick individuals chose not to seek care when they were ill. For example, hospitalization can cost up to nearly 7 times more than an impoverished rural residents’ annual income [2]. Therefore, reducing the large social disparity in healthcare utilization has become a key priority for the Chinese central government.

To achieve these goals, the Chinese central government has established a series of health reforms since the 20th century. Several indicators have shown the government’s efforts to improve its healthcare system. First, from the early 21st century to 2011, the health insurance coverage rate increased rapidly from approximately 21% and 56% in rural and urban areas, respectively, to nearly 95% in general [2,3,4]. The expansion of social insurance could provide basic coverage to uninsured individuals. Second, with the dramatic expansion of social insurance plans, the sharp decrease of the share of out-of-pocket expenses in total health expenditures was another critical factor. For example, out-of-pocket expenditure was approximately 60% in 2000 but fell to less than 30% in 2016 [5]. Meanwhile, the share of government spending on healthcare services increased from 15% to 30% between 2000 and 2015 [5]. The Chinese central government has made steady progress in improving its healthcare system, and it is expected to implement further reforms in the future.

With the improvements in healthcare and social insurance, China has two major systems targeting rural and urban populations with three social insurance schemes: (1) Urban Employment Basic Medical Insurance (UEBMI, for individuals who work for the formal or registered sectors), (2) Urban Resident Basic Medical Insurance (URBMI, for individuals who do not have a regular job, such as students or disabled individuals), and (3) the New Cooperative Medical Scheme (NCMS, for rural residents). China has planned to achieve genuinely universal coverage by 2020 and has attempted to merge the URBMI and NCMS schemes [6,7], in a step toward reducing social disparities among enrollees. Rural residents who migrated to urban areas could have greater financial flexibility to use healthcare services in urban regions when they are sick in urban areas. The latter might be one solution to reduce the social disparities between rural and urban areas.

Providing adequate healthcare services to individuals with most medical needs could contribute to more positive health outcomes. Hao et al. [8] found that receiving adequate healthcare services was associated with longer life expectancy among Chinese older adults. Receiving adequate healthcare services could make a difference in healthy longevity among Chinese older adults as well [9]. On the other hand, when individuals do not have sufficient healthcare services, the consequences also might be related to different health outcomes including psychological distress, poorer physical health, and a higher level of cognitive impairment [10,11]. In general, having adequate healthcare services is vital to maintaining healthy lifestyles in the older adult population.

However, this is easier said than done. China is a country with large social disparities such as urban and rural differences [11,12]. Chinese adults with a higher level of education and income had higher odds of using preventive care services, compared with those with lower levels of education and income [3,13]. Social gaps also exist across different social insurance schemes. Higher levels of education and income were positively associated with UEBMI coverage but negatively associated with the NCMS scheme [14]. As China aims to achieve genuine universal healthcare, the social insurance system should not have such social gaps.

Interestingly, in another study by Lee et al. [15], the empirical results showed that education was not associated with either urban scheme and was associated only with NCMS. However, household income was still an important factor associated with different types of social insurance coverage among Chinese older adults. All in all, with the benefits mentioned above of having adequate healthcare services, it is imperative to examine what socioeconomic factors might be associated with adequate healthcare services among Chinese older adults—a rapidly escalating population in China since the early 21st century [16].

This research aims to investigate the associations of socioeconomic status with receiving adequate healthcare services among Chinese older adults and identify vulnerable groups following the 2009 Chinese health reform. In this case, policy makers and scholars could use the empirical findings from this study to implement more effective policies and reduce the potential social gaps. Our research hypothesis suggests that socioeconomic status is associated with receiving adequate healthcare services among Chinese older adults. With the aforementioned literature, it is possible that older adults with higher socioeconomic status had higher chances of receiving adequate healthcare services, compared with more impoverished individuals. Further research directions and policy implications are discussed based on the empirical findings.

## 2. Materials and Methods

### 2.1. Study Sample

Secondary data were extracted from the 6th and 7th waves of the Chinese Longitudinal Healthy Longevity Survey (CLHLS) that were collected between 2011 and 2012 (6th wave), and in 2014 (7th wave), respectively. The 1st wave of CLHLS was collected in 1998. CLHLS is a large database established by the Center for the Study of Aging and Human Development at Duke University, together with other international collaborators. In this database, research participants include middle-aged adults 35 to 64 years old, younger older adults 65 to 79 years old, octogenarians, nonagenarians, and centenarians. Study participants were randomly selected from major provinces and mega cities. CLHLS investigators conducted data collection through face-to-face interviews. The CLHLS questionnaire includes a comprehensive array of topics such as social policy, family relationships, dietary behaviors, disability, mental health, substance use, and other topics related to older adults’ lifestyle and wellbeing. CLHLS is one of the first datasets examining older adults’ lifestyle, longevity, and healthy aging in China. Investigators obtained informed consent from all research participants. Zeng [17] provides more information about this dataset. The CLHLS dataset can be downloaded directly from the National Archive of Computerized Data on Aging (NACDA).

This research adopted a repeated cross-sectional study design. To create the study sample, data were analyzed from older adults who answered all questions of interest without missing responses. Older adults, 65 years old or above, were included. The 6th wave was treated as the baseline wave, given that it was the first wave available following the 2009 health reform in China. Furthermore, in the 7th wave, participants were included who did not participate in the previous survey. This method may help reduce the potential issues related to selection bias because repeated survey participation within a short time might lead to a higher awareness of using healthcare services. With the selection criteria, *n* = 9305 participants were obtained in the final study sample. As this research was based on a publicly available secondary dataset with de-identifiable information, this study did not constitute human subjects research (45 CFR 46.102), in which case it did not require Institutional Review Board (IRB) review.

### 2.2. Outcome Variable

According to the CLHLS questionnaire, there is one question asking when the participant received adequate healthcare services or treatments if they were seriously ill (no/yes; *“If you are seriously ill, can you get adequate healthcare service?”*; Chinese version: *“如果您生重病,请问能及时到医院治疗吗?”*). This was the outcome variable for the present study.

### 2.3. Socioeconomic Status

To conduct the secondary analysis, five measurements were selected to represent older adults’ socioeconomic status. The first measurement in this set of variables was the types of community (urban/rural). Participants who resided in cities and towns were categorized as “urban.” Others residing in smaller units were classified as “rural.” Next, for marital status (married/not married), older adults who were not married, widowed, separated, or divorced were coded as “not married.” Participants’ household income was divided into quintiles: 1st quintile of 10,000 RMB or lower, 2nd quintile of between 10,001 RMB and 30,000 RMB, 3rd quintile of between 30,001 RMB and 50,000 RMB, 4th quintile of between 50,001 RMB and 70,000 RMB, and 5th quintile of 70,001 RMB or above. Furthermore, older adults who did not know their household income were coded as a separate category.

One variable asking older adults’ educational background was selected, measured as years of formal education received. This variable was classified into “none,” “1 to 5 years,” “6 to 10 years,” and “11 years or above.” A major source of coverage for healthcare expenses was selected as the information regarding the primary financial source for medical bills including “UEBMI,” “URBMI,” “NCMS,” “Others,” “self-payment,” and “cannot afford to pay.” Unlike approaches that measure only coverage information, this variable examined the most frequently-used or relied-on coverage for healthcare expenses among older adults.

### 2.4. Control Variables

Two measurements were selected to describe older adults’ biological characteristics: gender (male/female) and age (65–80, 81–95, and above 95; measured in years).

Geographical region was included to assess the role of participants’ residential provinces when they joined the interview. Geographical region was classified into five categories: North—Tianjin, Hebei, Shanxi, and Beijing; Northeast—Liaoning, Jilin, and Heilongjiang; East—Jiangsu, Zhejiang, Anhui, Fujian, Jiangxi, Shandong, and Shanghai; Central-South—Hainan, Henan, Hunan, Hubei, Guangdong, and Guangxi; and West—Sichuan, Shaanxi, and Chongqing. This method of classification was based on guidelines from the CLHLS website (https://sites.duke.edu/centerforaging/programs/chinese-longitudinal-healthy-longevity-survey-clhls/project-goals/coverage-of-sampled-provinces/).

Next, a set of variables was selected to describe older adults’ health behaviors, wellbeing, and health status. The number of times suffering from chronic diseases in the past two years was selected to indicate older adults’ chronic conditions (none, 1 to 2 times, and above 2 times). Life satisfaction was selected to describe participants’ basic wellbeing (“very good,” “good,” “neutral,” “bad,” and “very bad”). Two variables were selected regarding older adults’ substance use information: current alcohol use (no/yes) and current smoking status (no/yes). Current exercise status (no/yes) was used to examine older adults’ physical activity.

Table 1 shows the list of selected variables in this research.

### 2.5. Statistical Analysis

Three Firth’s logistic regression models were fitted (since the outcome variable is binary). Firth’s logistic regression is a type of penalized logistic regressions if the outcome is imbalanced [18]. In our case, according to the descriptive statistics, only 5.3% of the participants cannot receive adequate healthcare services if they are seriously ill. Therefore, using Firth’s logistic regression was necessary in this research. Model 1 controlled for biological factors and health-related measurements; model 2 controlled for biological variables and socioeconomic status; and model 3 was a full model with biological variables, health-related measurements, and socioeconomic status. Three separate regression models were estimated, to investigate differences among selected variables in this research. Furthermore, we used the adjusted generalized variance inflation factor (AGVIF) to examine potential issues related to multicollinearity. To prevent potential issues related to multicollinearity in the statistical analysis, all values from AGVIF should be smaller than two (AGVIF <2). In our preliminary results, all calculated values were smaller than two.

Adjusted odds ratio (AOR) and 95% confidence interval (95% CI) were reported to summarize the results of the regressions. Chi-square tests also were performed to examine correlations between each predictor and the outcome. All regression results were two-sided with level of significance of 0.05 (*p* < 0.05), using the free and publicly available statistical software R (version 3.6.2). The R package “brglm2” was used to carry out the primary analysis [18].

## 3. Results

### 3.1. Descriptive Statistics

Table 2 shows the descriptive statistics of the final study sample. The majority of participants (94.7%) claimed adequate healthcare services if they were seriously ill. Only 5.3% of the older adults did not claim adequate healthcare services. The majority of the participants were female, below 95 years old, and not married. Most participants did not have any chronic diseases in the past two years (79.4%), did not smoke, did not consume alcohol, and did not exercise. Most participants reported “good” and “very good” for life satisfaction and resided in rural areas. In terms of geographical regions, the majority of the participants resided in the East and the Central-South. Approximately 5.6% of older adults did not receive adequate healthcare services in 2012, but this number dropped to just 3.5% in 2014.

### 3.2. Model 1

Table 3 shows the results of Firth’s logistic regression models for receiving adequate healthcare services if the participants are seriously ill (Models 1 to 3). The first model controlled for the biological and health-related variables. Older age groups were less likely to report receiving adequate healthcare services (all *p*s < 0.01). Older adults who reported lower levels of life satisfaction also were less likely to report receiving adequate healthcare services (all *ps* < 0.01). Older adults who exercised had higher odds of receiving adequate healthcare services (AOR = 1.86, 95% CI: 1.47, 2.35; *p* < 0.01), compared with those who did not exercise. Current smokers were less likely to receive adequate healthcare services (AOR = 0.60, 95% CI: 0.47, 0.78; *p* < 0.01).

### 3.3. Model 2

The second model adjusted for biological variables and variables related to older adults’ socioeconomic status. In this model, gender and age were not associated with adequate healthcare services. Non-married participants had lower odds of receiving adequate healthcare services (AOR = 0.67, 95% CI: 0.53, 0.84; *p* < 0.01), compared with married participants. Older adults with higher levels of household income categories were positively associated with adequate healthcare services (all *p*s < 0.01). Participants who relied on non-urban insurance schemes were less likely to receive adequate healthcare services (all *p*s < 0.01). Older adults with 11 years of formal education or above were positively associated with receiving adequate healthcare service (AOR = 5.35, 95% CI: 1.09, 26.36; *p* < 0.05). The differences between urban and rural areas were not significantly associated with adequate healthcare services.

### 3.4. Model 3

The third model controlled for all variables included in this research. Lower levels of life satisfaction were negatively associated with receiving adequate healthcare services (all *p*s < 0.05), except for the difference between “good” and “very good.” Older adults who exercised had higher odds of receiving adequate healthcare services (AOR = 1.54, 95% CI: 1.21, 1.95; *p* < 0.01), compared with participants who did not exercise. Being non-married was negatively associated with receiving adequate healthcare services (*p* < 0.01). Higher-income was positively associated with receiving adequate healthcare services (all *p*s < 0.05). Older adults who used non-urban insurance schemes were less likely to receive adequate healthcare services (all *p*s < 0.01). Again, the differences between urban and rural areas were not significantly associated with adequate healthcare services. Education was not associated with adequate healthcare service utilization. Among the three models, the 2014 wave was positively associated with adequate healthcare services (all *p*s < 0.01).

## 4. Discussion

### 4.1. General Discussion

This study investigated the wealth gap in receiving adequate healthcare services among Chinese older adults, if they are seriously ill. This secondary analysis of survey data was based on one of the unique large databases targeting Chinese older adults [17]. The study design made it possible to examine the most important variables associated with receiving adequate healthcare services and identify economically disadvantaged older adults who might need further assistance.

Surprisingly, based on the descriptive statistics, only 5.3% of the study participants reported they did not receive adequate healthcare services if they were seriously ill. Also, in just the two years from 2012 to 2014, the percentage of older adults who did not receive adequate healthcare services dropped from 5.6% to 3.5%. In the same vein, the percentage of patients who needed healthcare services but could not afford them financially dropped from 17.6% to 7.4% between 2008 and 2013 [19]. The increased affordability of healthcare services could be the result of coverage expansion and growing availability of healthcare services [20]. These variables might explain why nearly 95% of the older adults included in this study claimed that they received adequate healthcare services if they needed them following the 2009 health reform.

The estimates of Firth’s logistic regression parameters in models 2 and 3 demonstrated that older adults with higher household income had higher odds of receiving adequate healthcare services, compared to individuals with the lowest level of income. Participants with non-urban social insurance schemes had lower odds of receiving adequate healthcare services, compared with older adults with UEBMI coverage. In Model 3, adjusted for all variables, higher income was positively associated with receiving adequate healthcare services. Interestingly, older adults’ educational background was neither positively nor negatively associated with receiving adequate healthcare services in the fully adjusted model, which is Model 3 in this analysis.

### 4.2. Education and Income

In this present research, both knowledge gaps and similarities were observed for educational background. These findings regarding the insignificant association of education contradicted the traditional belief that a higher level of education might indicate better health or healthier behavior. For example, in Wisconsin, a Mid-Western state in the United States, attending college was associated with higher odds of using preventive care services such as physical examinations, cholesterol tests, and flu shots [21]. Besides, compared with individuals with lower levels of education, adults with higher levels of education usually live healthier and longer lives [22]. Education is not only a primary source of better health, but it is also considered a long-term cause of positive health outcomes [23]. Nevertheless, these benefits of having a higher level of education were not observed in this research when controlling for all covariates (Model 3).

The insignificant association in the full model between older adults’ education and the utilization of healthcare services might be explained in different ways. The only exception was the association between the highest level of education (11 years or above) and no education in model 2, adjusted for only biological variables and wealth attainment. First, based on descriptive statistics, nearly 60% of the participants received no education. Therefore, the comparisons among different categories may not be statistically significant due to a smaller numbers of observations such as older adults with the highest level of education. Second, it is reasonable to speculate that individuals with higher levels of education might have higher awareness of personal health. For example, Chinese older adults with primary school education had lower mean health knowledge and behaviors than those with high school and college education [24]. Highly-educated individuals might use more preventive care services and have healthier behaviors to maintain their health condition [3,24]. Because our research outcome measurement asked participants if they received adequate healthcare services if they were ill, older adults with higher levels of education and better health awareness may not need healthcare services at all, if they were healthy. Third, China did not revamp the education system until the late 1970s, together with its economic reform and open-door policy [25]. The older participants included in our study sample did not experience such educational reform. Thus, the education they received when they were younger may not represent current educational disparity in China. With these knowledge gaps, the educational disparity in inadequate utilization of the healthcare service should be studied further, given that education is one of the best predictors of health outcomes [26].

The observations of income gaps in receiving adequate healthcare service were not as surprising. Income has been considered an important variable associated with health because it provides an opportunity to exercise control over one’s life including material deprivation and restrictions on social participation [27]. For example, although Taiwan’s National Health Insurance (NHI) covers approximately 99% of the population [28], insured individuals need to pay out-of-pocket expenses to cover extra medical bills such as physical examination or hospitalization. Therefore, poorer individuals cannot pay for large medical expenses, lowering the odds of having adequate healthcare services. A similar situation might occur in China because China’s universal healthcare is still at an early stage. In China, income disparity is evident, even with different types of social insurance schemes [15].

Additionally, China is a country known for its large social inequalities, and income is one critical element in poverty, social disparities, and threats to health [29,30]. In rural China, wealthier counties have more health resources and hospitalizations; furthermore, absolute inequalities in healthcare resources also increased over time [31]. Even after the 2009 health reform aiming at inequality reduction, not having a higher level of income is still a barrier thwarting preventive care utilization among Chinese citizens [13]. These observations of the income gap might suggest that the 2009 health reform may not have been very successful in bringing more equitable outcomes for healthcare utilization in China. Thus, as further health reforms unfold, it is imperative to reduce income disparity within the Chinese healthcare system.

### 4.3. Marital Status

It is widely believed that older adults who live alone (i.e., unmarried individuals) have higher chances of neglecting their health status. For example, if unmarried older adults are already sick but do not have the ability or knowledge to recognize their health condition, they can miss early opportunities to receive treatment. When they notice their worsening health, their condition might be worse than if they had received an early diagnosis. Compared with unmarried older adults, married older adults usually have support from their partner or children to monitor their health. Under such circumstance, the chances of receiving early treatment for a disease also might be higher. In previous research, the importance of marriage has been observed such as lower levels of depression, higher life satisfaction, and better health status [32,33,34]. In this study, unmarried individuals had lower odds of receiving adequate healthcare services if they were ill. Therefore, social support from the local community should target these unmarried older adults, given that they might be financially disadvantaged without family assistance and thus might neglect their health.

### 4.4. Primary Coverage for Healthcare Expenses and Community Disparity

Community disparity is linked with primary coverage for healthcare expenses due to observable gaps between urban and rural areas. In this research, it is interesting that statistically significant associations between types of community and utilization of adequate healthcare services were not found in models 2 and 3. However, the large urban and rural disparities continue to affect healthcare utilization and health status in general [35,36]. Nevertheless, the comparisons between NCMS and UEBMI were statistically significant (all *p*s < 0.01). Additionally, we did not find statistically significant differences between the two urban schemes, URBMI and UEBMI. In models 2 and 3, non-urban social insurance plans or different sources of coverage all were negatively associated with receiving adequate healthcare services.

These observations might be explained in different ways. First, URBMI and UEBMI cover urban residents [1], but the urban enrollees might have similar healthcare resources, regardless of the differences between URBMI and UEBMI. Although URBMI has shallower benefits covering healthcare expenses following the 2009 health reform [37], rural residents usually underutilize healthcare services compared with urban residents [35]. For example, urban adults were 2.23 and 4.77 times more likely to use outpatient and inpatient care, respectively, compared with their rural counterparts [35]. Moreover, the social disparities across UEBMI, URBMI, and NCMS have been well-documented [14]. Hence, the primary coverage of healthcare services, including the significant difference between NCMS and UEBMI and the lack of a significant difference between URBMI and UEBMI, might be the better indicator to examine the actual urban-rural disparities than types of community.

Although we did not find a statistically significant difference between urban and rural areas, the discrepancy between NCMS and UEBMI might suggest potential urban–rural disparity. Several indicators could explain this discrepancy. Rural residents not only have a shortage of healthcare providers; they also have lower socioeconomic status and limited social support [35]. In addition, because NCMS includes shallower benefits to cover the large medical bills as compared with UEBMI, sick rural patients may not be able to receive the care they need. On the other hand, registered doctors per thousand populations in urban areas were almost 2.6 times more available than doctors in rural areas [35]. There are still more medical staff and resources in urban areas than in rural areas.

Also, nearly 58% of the participants relied on self-payment as the major source to cover their medical expenses. The latter might suggest that the social insurance schemes still could not become the most reliable coverage for medical expenses. Specifically, when older adults relied on self-payment, they had lower odds of receiving adequate healthcare services if they were ill. In other words, they may not seek necessary care if they were sick due to financial hurdles. Even with the nearly universal coverage of social insurance in China [1,2], these schemes may not provide sufficient financial benefits to cover the sick and insured individuals. This might indicate the situation of “coverage without access” in China; insured individuals might have a specific type of coverage, but they still cannot use these insurance plans effectively to cover necessary medical expenses. When poor people pay a greater share of out-of-pocket expenses for medical bills, their chances of utilizing healthcare services could be lower. Making social insurance the reliable form of coverage for medical expenses should be a key for the Chinese central government in the near future.

Nevertheless, less than 1% of older adults claimed that they were not able to pay for healthcare expenses. Of course, these individuals also had lower odds of receiving adequate healthcare services. Although this number is fractional (less than 1%), further public health efforts and policy implementation need to target these poorer older adults and provide more assistance for receiving essential healthcare services.

### 4.5. Limitations

This research is not without limitations. First, as the CLHLS dataset includes only two waves of surveys following the 2009 health reform, it was not possible to investigate the long-term trends of adequate healthcare services among older adults. Nevertheless, as previously mentioned, it is worth pointing out that the percentage of older adults who did not receive adequate healthcare services declined by approximately 2% between the two waves. Therefore, this percentage might continue to decline in the foreseeable future with further reforms underway. Second, this research relied on a secondary dataset. Self-reporting bias or recall bias might occur (e.g., the ability to recall previously-reported household income). However, this is a common limitation for most survey-based research, and should not be a primary concern for this study. Third, as China already has planned to consolidate URBMI and NCMS as one universal scheme, this analysis can show results only before the consolidation. Further research with similar interests should attempt to resolve this study limitation. Finally, it was not possible to determine whether older adults received better healthcare services in local clinics or large medical centers due to the CLHLS questionnaire design. We were not able to investigate the accessibility or availability of healthcare services in different locations. Different places to seek healthcare services might affect older adults’ decision making.

## 5. Conclusions

Despite these limitations, this study adds to the body of literature examining the associations of socioeconomic status with adequate healthcare services among older adults following the 2009 health reform in China. Our study confirmed that participants with higher income were more likely to receive adequate healthcare services. However, older adults with non-urban social insurance plans were less likely to achieve adequate utilization of healthcare services. Non-married individuals also had lower odds of receiving adequate healthcare services, compared with married participants. Surprisingly, we did not find disparities between urban and rural areas or an association of disparities with educational achievement in the fully adjusted model (Model 3). Further research should continue to investigate the urban–rural disparities and the association of education with receiving adequate healthcare services. As the Chinese central government is pushing further reforms, policy makers should target vulnerable older adults such as providing further financial benefits to older adults living with lower levels of income. Furthermore, the differences among UEBMI, NCMS, and other types of coverage for healthcare expenses should be investigated further. Although China has planned to consolidate NCMS and URBMI as one major scheme, the differences between the consolidated scheme and UEBMI with the utilization of adequate healthcare services warrant more research efforts.

## Figures and Tables

**Table 1:** Selected control variables for the final study sample.

Biological Factors	Socioeconomic Status	Health-Related Measurements	Other Measurements
1. Gender (a binary variable) 2. Age (a categorical variable)	1. Community (a binary variable) 2. Marital status (a binary variable) 3. Household income (a categorical variable) 4. Years of formal education (a categorical variable) 5. Major source of coverage for healthcare expenses (a categorical variable)	1. Number of times suffering from chronic diseases in the past two years (a categorical variable) 2. Life satisfaction (a categorical variable) 3. Current alcohol use (a binary variable) 4. Current smoking status (a binary variable) 5. Current exercise status (a binary variable)	1. Wave (a binary variable) 2. Geographical region (a categorical variable)

**Table 2:** Descriptive statistics of the final study sample: Chinese Longitudinal Healthy Longevity Survey (CLHLS), 2012–2014 (*n* = 9305).

Measurements	Distribution		Receive Adequate Healthcare Services if They are Seriously Ill	
*Biological factors:*	Overall (*n*, %)		No (*n*, %)	Yes (*n*, %)
	*n* = 9305 (100%)	*p-value*	*n* = 493 (5.3%)	*n* = 8812 (94.7%)
			
Gender		0.054		
Male	4279 (46%)		206 (4.8%)	4073 (95.2%)
Female	5026 (54%)		287 (5.7%)	4739 (94.3%)
Age (in years)		<0.001		
65–80	3443 (37%)		143 (4.2%)	3300 (95.8%)
81–95	4054 (43.6%)		233 (5.7%)	3821 (94.3%)
Above 95	1808 (19.4%)		117 (6.5%)	1691 (93.5%)
*Health-related measurements:*				
Number of times suffering from chronic diseases in the past two years		0.188		
None	7392 (79.4%)		407 (5.5%)	6985 (94.5%)
1 to 2 times	1682 (18.1%)		74 (4.4%)	1608 (95.6%)
Above 2 times	231 (2.5%)		12 (5.2%)	219 (94.8%)
Life satisfaction	1542 (16.6%)	<0.001	25 (1.6%)	1517 (98.4%)
Very good				
Good	4204 (45.2%)		148 (3.5%)	4056 (96.5%)
Neutral	3082 (33.1%)		202 (6.6%)	2880 (93.4%)
Bad	413 (4.4%)		97 (23.5%)	316 (76.5%)
Very bad	64 (0.7%)		21 (32.8%)	43 (67.2%)
Current alcohol use		0.117		
No	7705 (82.8%)		421 (5.5%)	7284 (94.5%)
Yes	1600 (17.2%)		72 (4.5%)	1528 (95.5%)
Current smoking status		0.025		
No	7602 (81.7%)		384 (5.1%)	7218 (94.9%)
Yes	1703 (18.3%)		109 (6.4%)	1594 (93.6%)
Current exercise status		<0.001		
No	6172 (66.3%)		395 (6.4%)	5777 (93.6%)
Yes	3133 (33.7%)		98 (3.1%)	3035 (96.9%)
*Socioeconomic status:*				
Community		<0.001		
Urban	4329 (46.5%)		177 (4.1%)	4152 (95.9%)
Rural	4976 (53.5%)		316 (6.4%)	4660 (93.6%)
Marital status		<0.001		
Married	3688 (39.6%)		138 (3.7%)	3550 (96.3%)
Not married	5617 (60.4%)		355 (6.3%)	5262 (93.7%)
Household income (in quintiles)		<0.001		
1	3672 (39.5%)		330 (9%)	3342 (91%)
2	2642 (28.4%)		95 (3.6%)	2547 (96.4%)
3	1191 (12.8%)		17 (1.4%)	1174 (98.6%)
4	434 (4.7%)		8 (1.8%)	426 (98.2%)
5	726 (7.8%)		3 (0.4%)	723 (99.6%)
Do not know	640 (6.9%)		40 (6.2%)	600 (93.8%)
Years of formal education		<0.001		
None	5336 (57.3%)		334 (6.3%)	5002 (93.7%)
1 to 5	2224 (23.9%)		110 (4.9%)	2114 (95.1%)
6 to 10	1387 (14.9%)		48 (3.5%)	1339 (96.5%)
11 or above	358 (3.8%)		1 (0.3%)	357 (99.7%)
Major source of coverage for healthcare expenses		<0.001		
Urban Employment Basic Medical Insurance (UEBMI)	975 (10.5%)		5 (0.5%)	970 (99.5%)
Urban Resident Basic Medical Insurance (URBMI)	351 (3.8%)		7 (2%)	344 (98%)
New Cooperative Medical Scheme (NCMS)	2353 (25.3%)		136 (5.8%)	2217 (94.2%)
Others	209 (2.2%)		17 (8.1%)	192 (91.9%)
Self-payment	5386 (57.9%)		313 (5.8%)	5073 (94.2%)
Can’t afford to pay	31 (0.3%)		15 (48.4%)	16 (51.6%)
Wave		0.002		
2012	8078 (86.8%)		450 (5.6%)	7628 (94.4%)
2014	1227 (13.2%)		43 (3.5%)	1184 (96.5%)
Geographical region		<0.001		
North	313 (3.4%)		5 (1.6%)	308 (98.4%)
Northeast	501 (5.4%)		20 (4%)	481 (96%)
East	3557 (38.2%)		142 (4%)	3415 (96%)
Central-South	3870 (41.6%)		259 (6.7%)	3611 (93.3%)
West	1064 (11.4%)		67 (6.3%)	997 (93.7%)

**Table 3:** Results of Firth’s logistic regressions: Chinese Longitudinal Healthy Longevity Survey (CLHLS), 2012–2014.

Measurements	Model 1	Model 2	Model 3
*Biological factors:*	AOR^a^ (95% CI)^b^	AOR (95% CI)	AOR (95% CI)
		
Gender			
Male	--		
Female	0.82 (0.66,1.02)	1.12 (0.90,1.39)	0.97 (0.76,1.23)
Age (in years)			
65–80			
81–95	0.72 (0.58,0.90) **	0.83 (0.66,1.05)	0.80 (0.63,1.02)
Above 95	0.63 (0.48,0.82) **	0.81 (0.61,1.08)	0.72 (0.54,0.97) *
*Health-related measurements:*			
Number of times suffering from chronic diseases in the past two years			
None			
1 to 2 times	1.32 (1.02,1.72) *		1.17 (0.90,1.52)
Above 2 times	1.18 (0.65,2.16)		0.98 (0.53,1.80)
Life satisfaction			
Very good			
Good	0.54 (0.35,0.82) **		0.65 (0.43,1.00)
Neutral	0.29 (0.19,0.44) **		0.39 (0.25,0.59) **
Bad	0.07 (0.04,0.10) **		0.11 (0.07,0.17) **
Very bad	0.04 (0.02,0.07) **		0.07 (0.03,0.13) **
Current alcohol use			
No			
Yes	1.20 (0.91,1.59)		1.20 (0.90,1.59)
Current smoking status			
No			
Yes	0.60 (0.47,0.78) **		0.64 (0.49,0.83) **
Current exercise status			
No			
Yes	1.86 (1.47,2.35) **		1.54 (1.21,1.95) **
*Socioeconomic status:*			
Community			
Urban			
Rural		0.88 (0.72,1.08)	0.91 (0.74,1.12)
Marital status			
Married			
Not married		0.67 (0.53,0.84) **	0.68 (0.54,0.86) **
Household income (in quintiles)			
1			
2		2.35 (1.86,2.98) **	1.98 (1.55,2.51) **
3		4.94 (3.04,8.04) **	3.94 (2.42,6.43) **
4		3.12 (1.57,6.22) **	2.24 (1.12,4.49) *
5		13.62 (4.78,38.80) **	9.05 (3.18,25.72) **
Do not know		1.48 (1.05,2.10) *	1.33 (0.93,1.90)
Years of formal education			
None			
1 to 5		1.07 (0.83,1.37)	0.97 (0.75,1.26)
6 to 10		1.19 (0.84,1.68)	1.05 (0.74,1.49)
11 or above		5.35 (1.09,26.36) *	4.07 (0.82,20.07)
Major source of coverage for healthcare expenses			
UEBMI			
URBMI		0.43 (0.14,1.28)	0.40 (0.14,1.21)
NCMS		0.25 (0.11,0.59) **	0.27 (0.12,0.64) **
Others		0.15 (0.06,0.39) **	0.18 (0.07,0.48) **
Self-payment		0.25 (0.11,0.57) **	0.28 (0.12,0.66) **
Can’t afford to pay		0.02 (0.01,0.07) **	0.04 (0.01,0.14) **
Wave			
2012			
2014	1.71 (1.23,2.37) **	1.62 (1.17,2.25) **	1.57 (1.13,2.20) **
Geographical region			
North			
Northeast	0.46 (0.17,1.21)	0.49 (0.19,1.27)	0.52 (0.19,1.37)
East	0.49 (0.21,1.19)	0.67 (0.28,1.58)	0.65 (0.27,1.57)
Central	0.35 (0.15,0.84) *	0.44 (0.19,1.04)	0.52 (0.22,1.25)
West	0.31 (0.13,0.76) *	0.42 (0.17,1.00)	0.40 (0.16,0.99) *

* *p* < 0.05; ** *p* < 0.01; Model 1: Biological factors + health-related measurements; Model 2: Biological factors + socioeconomic status; Model 3: Biological factors + health-related measurements + socioeconomic status. ^a^ AOR = Adjusted odds ratio; ^b^ 95% CI = 95% Confidence Interval.
